# Identification of core genes in intervertebral disc degeneration using bioinformatics and machine learning algorithms

**DOI:** 10.3389/fimmu.2024.1401957

**Published:** 2024-07-10

**Authors:** Hao Zhang, Shengbo Shi, Xingxing Huang, Changsheng Gong, Zijing Zhang, Zetian Zhao, Junxiao Gao, Meng Zhang, Xiaobing Yu

**Affiliations:** Department of Orthopaedics, Affiliated Zhongshan Hospital of Dalian University, Dalian, Liaoning, China

**Keywords:** intervertebral disc degeneratio1, bioinformatics, machine learning, genes, IL1R15, TCF7L2

## Abstract

**Background:**

Intervertebral Disc Degeneration (IDD) is a major cause of lower back pain and a significant global health issue. However, the specific mechanisms of IDD remain unclear. This study aims to identify key genes and pathways associated with IDD using bioinformatics and machine learning algorithms.

**Methods:**

Gene expression profiles, including those from 35 LDH patients and 43 healthy volunteers, were downloaded from the GEO database (GSE124272, GSE150408, GSE23130, GSE153761). After merging four microarray datasets, differentially expressed genes (DEGs) were selected for GO and KEGG pathway enrichment analysis. Weighted Gene Co-expression Network Analysis (WGCNA) was then applied to the merged dataset to identify relevant modules and intersect with DEGs to discover candidate genes with diagnostic value. A LASSO model was established to select appropriate genes, and ROC curves were drawn to elucidate the diagnostic value of genetic markers. A Protein-Protein Interaction (PPI) network was constructed and visualized to determine central genes, followed by external validation using qRT-PCR.

**Results:**

Differential analysis of the preprocessed dataset identified 244 genes, including 183 upregulated and 61 downregulated genes. WGCNA analysis revealed the most relevant module intersecting with DEGs, yielding 9 candidate genes. The lasso-cox method was used for regression analysis, ultimately identifying 6 genes: ASPH, CDC42EP3, FOSL2, IL1R1, NFKBIZ, TCF7L2. A Protein-Protein Interaction (PPI) network created with GENEMANIA identified IL1R1 and TCF7L2 as central genes.

**Conclusion:**

Our study shows that IL1R1 and TCF7L2 are the core genes of IDD, offering new insights into the pathogenesis and therapeutic development of IDD.

## 1 Introduction

Lower back pain is one of the most common medical issues worldwide, affecting 70% to 85% of the population globally (1). Intervertebral Disc Degeneration (IDD) is the primary cause of lower back pain (2). Currently, the treatment strategies for IDD mainly include conservative and surgical treatments, but these methods often only alleviate pain without halting the progression of IDD (3). Surgical treatment can have long-term adverse effects on biomechanics, while conservative treatment is usually temporary (4). IDD is a complex, multifactorial process involving changes in phenotype and genotype, hence, gene-targeted therapy may be a promising direction for future IDD treatments (5). Recent studies have found that immune cell infiltration and the increase in inflammatory mediators and oxidative stress play a crucial role in the development of IDD, accelerating the inflammatory cascade and ultimately leading to discogenic pain (6). Therefore, a deeper understanding of the pathological changes and mechanisms of IDD is essential for diagnosis and the development of new treatment methods.

Bioinformatics, an emerging biotechnology that has grown with the Human Genome Project, utilizes computer technology to store, retrieve, analyze, and interpret large volumes of biological data (7). In the last two decades, with the advent of the big data era and the continuous growth and inherent complexity of biological data, machine learning, as an interdisciplinary field, has been widely applied to construct informational models and predictions of biological processes, significantly impacting the field of biology.

The aim of this study is to identify core genes associated with IDD by analyzing RNA expression profiles from the Gene Expression Omnibus (GEO) database and to validate these genes through *in vitro* experiments, with the goal of establishing these core genes as potential biomarkers for IDD.

## 2 Methods

### 2.1 Collection and preprocessing of microarray data

This study downloaded four gene expression profile datasets related to Intervertebral Disc Degeneration (IDD) from the Gene Expression Omnibus (GEO) database. The datasets include: GSE124272 (GPL21185), GSE153761 (GPL22120), GSE150408 (GPL21185), and GSE23130 (GPL1352), covering gene expression data from patients with lumbar disc herniation, cervical spondylotic myelopathy, and lumbar disc degeneration, as well as healthy volunteers. Data grouping was based on the Pfirrmann grading system (8), with grades I-III as the control group and grades IV-V as the experimental group. Initially, the R package inSilicoMerging was used to merge these datasets, followed by the application of a method proposed by Johnson et al (9). to remove batch effects, resulting in a corrected data matrix containing samples from 35 experimental and 43 control group participants.

### 2.2 Selection of differentially expressed genes

After merging the four datasets, further correction and normalization of the expression matrix were performed. Differential analysis was conducted using the Limma R package, with |log2FC| > 1.3 and p < 0.05 set as the criteria for selecting differentially expressed genes (DEGs). Subsequently, heatmaps and volcano plots were utilized to visually display the expression patterns of DEGs.

### 2.3 GO and KEGG analysis

Gene Ontology (GO) enrichment analysis and Kyoto Encyclopedia of Genes and Genomes (KEGG) pathway analysis were conducted to systematically analyze gene functions and key biological pathways closely related to Intervertebral Disc Degeneration (10). GO analysis results were categorized into Biological Processes (BP), Cellular Components (CC), and Molecular Functions (MF) (11). Enrichment analysis was performed using the DAVID database to identify statistically significant GO terms and KEGG pathways, with a selection threshold set at p < 0.05. All results were visualized using the ggplot2 R package.

### 2.4 WGCNA analysis

Weighted Gene Co-expression Network Analysis (WGCNA) was performed using the WGCNA package in R. Initially, outlier data were filtered to enhance model stability, and an appropriate soft threshold β was selected to construct the Topological Overlap Matrix (TOM). Hierarchical clustering methods were then used to generate a dendrogram of genes, and gene significance (GS) and module membership (MM) were calculated to assess the correlation between genes and clinical information, as well as to analyze the significant association between modules and disease characteristics.

### 2.5 Identification of characteristic genes and application of machine learning algorithms

Characteristic module genes were identified through cross-analysis with differentially expressed genes (DEGs) from the WGCNA analysis. Subsequently, the glmnet package in R was used to implement the LASSO machine learning algorithm, selecting significant genes with the highest predictive value for IDD. LASSO regression aims to simplify high-dimensional data by selecting characteristic genes with non-zero coefficients, followed by multivariate logistic regression analysis to identify all significant risk DEGs.

### 2.6 Construction of protein-protein interaction networks

The GeneMANIA network platform was utilized to construct Protein-Protein Interaction (PPI) networks to explore interactions and functions among core genes. GeneMANIA aids in the analysis and understanding of PPI networks by generating gene function predictions and identifying genes with similar functions (12).

### 2.7 Clinical diagnostic value assessment of core genes

Receiver Operating Characteristic (ROC) curves, drawn using the “pROC” package in R, were employed to evaluate the efficacy of core genes in the diagnosis of IDD. ROC curves depict the relationship between sensitivity and specificity, estimating the clinical diagnostic value of core genes by calculating the area under the curve (AUC).

### 2.8 Sample collection

This study protocol was approved by the Ethics Committee of Zhongshan Hospital affiliated with Dalian University. All participating patients signed informed consent forms. The severity of IDD in patients was assessed using magnetic resonance imaging (MRI) according to the Pfirrmann grading system (13). Intervertebral disc tissue samples were obtained from patients undergoing surgery for lumbar disc herniation.Finally, two patients with Pfirrmann grades III and V were selected. After the interdisc tissue was obtained, it was washed with phosphate buffer (PBS) to remove the impurities completely. The nucleus pulposum tissue was cut into 1mm fragments, digested with 0.2% type II collagenase, and incubated in a 37° incubator for digestion and incubation for 2 hours. The digestion was stopped with fetal bovine serum and phosphate buffer (PBS) containing more than 20% by volume, and then centrifuged. The superserum was discarded, transferred to dmem medium containing 10% fetal bovine serum and 1% penicillin streptomycin, and cultured in a co2 incubator. After 3 days of inoculation, the cell morphology of nucleus pulposum cells was observed with an inverted microscope. If the fusion degree reached 80%-90%, the subculture was performed again. Repeat this step to pass to the third generation.

### 2.9 Real-time quantitative polymerase chain reaction

Total RNA was extracted from nucleus pulposum tissues using StradyPure Universal RNA extraction kit, then transcribed into cDNA using the Cofitt Reverse Transcription Kit. The expression levels of target genes were determined using a high-specificity qRT-PCR detection kit, with specific PCR primer sequences including IL1R1 and TCF7L2.

### 2.10 Statistical analysis

Data processing and chart generation were performed using R 4.2.1 and GraphPad Prism 9 software. Statistical evaluation of the data was conducted using one-way ANOVA in SPSS 25.0 software, with differences considered statistically significant at P < 0.05.

## 3 Results

### 3.1 Preprocessing and selection of differentially expressed genes

**Figure 1:**
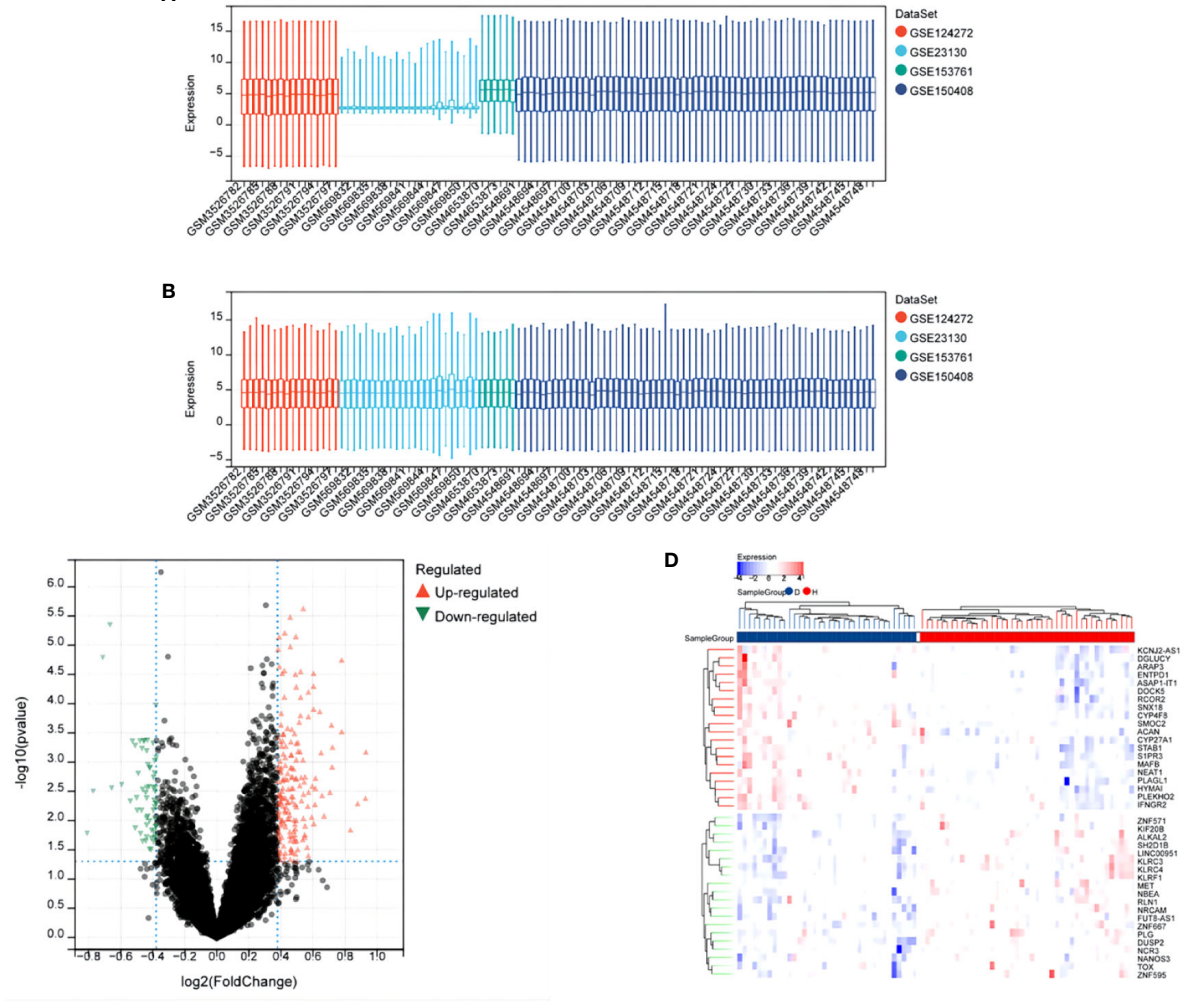
Differential Expression Analysis After Merging Datasets GSE124272, GSE153761, GSE150408, GSE23130. **(A)** Box plot of gene expression before batch effect removal. **(B)** Box plot of gene expression after batch effect removal. **(C)** Volcano plot of DEGs, x-axis: log2FoldChange, y-axis: -log10(adjusted p-value); red, black, and green dots represent upregulated, non-significant, and downregulated differentially expressed genes, respectively. **(D)** Heatmap showing significant differential expression of 40 genes (adjusted p-value ≤ 0.05). Red and blue indicate upregulated and downregulated genes, respectively.

After merging all datasets and removing batch effects, the data were normalized. The results showed that, after processing, the average expression levels of genes across the datasets were consistent (see **Figures 1A, B**). Differential analysis revealed a total of 244 differentially expressed genes (DEGs), including 183 upregulated and 61 downregulated genes. These results were visually displayed using a volcano plot (see **Figure 1C**). Additionally, the expression patterns of the identified DEGs were further presented using a heatmap (see **Figure 1D**).

### 3.2 Gene ontology enrichment analysis

**Figure 2:**
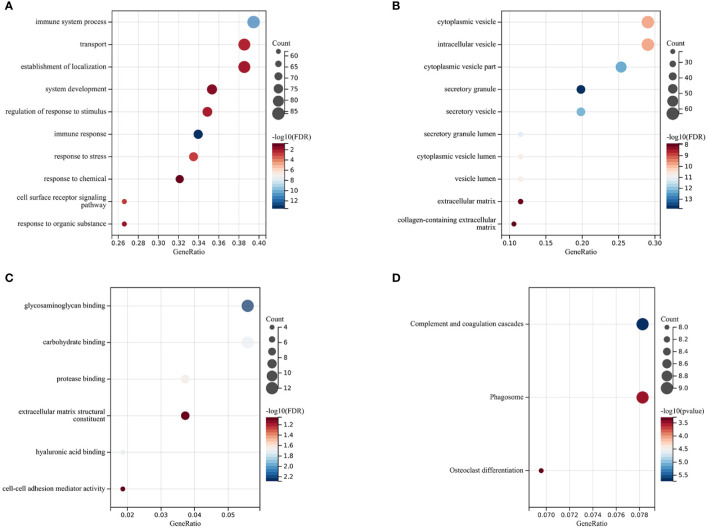
Functional Enrichment and KEGG Analysis The size of the circles represents the number of DEGs (the larger the circle, the more DEGs), and the color indicates the p-value (the redder, the smaller the value). **(A)** Biological Processes (BP). **(B)** Cellular Components (CC). **(C)** Molecular Functions (MF). **(D)** Kyoto Encyclopedia of Genes and Genomes (KEGG) Enrichment Analysis.

The Gene Ontology (GO) enrichment analysis revealed three main categories: Biological Processes (BP), Cellular Components (CC), and Molecular Functions (MF), selecting GO terms with a p-value of less than 0.05 and ranking them based on the number of involved genes (see **Figures 2A–C**). In the category of Biological Processes, a total of 10 GO terms were enriched, mainly involving immune system processes, developmental processes, response to stimulus, and response to stress. In the category of Cellular Components, 10 main GO terms were identified, including endomembrane systems, vesicles, extracellular regions, and intrinsic components of membranes. In the category of Molecular Functions, 6 items were enriched, covering glycosaminoglycan binding, carbohydrate binding, protease binding, extracellular matrix structural constituents, hyaluronic acid binding, and cell-cell adhesion mediator activity. KEGG enrichment analysis showed that differentially expressed genes (DEGs) are highly related to complement and coagulation cascades, phagosome pathways, and osteoclast differentiation pathways, with specific results shown in **Figure 2D**).

### 3.3 WGCNA analysis

**Figure 3:**
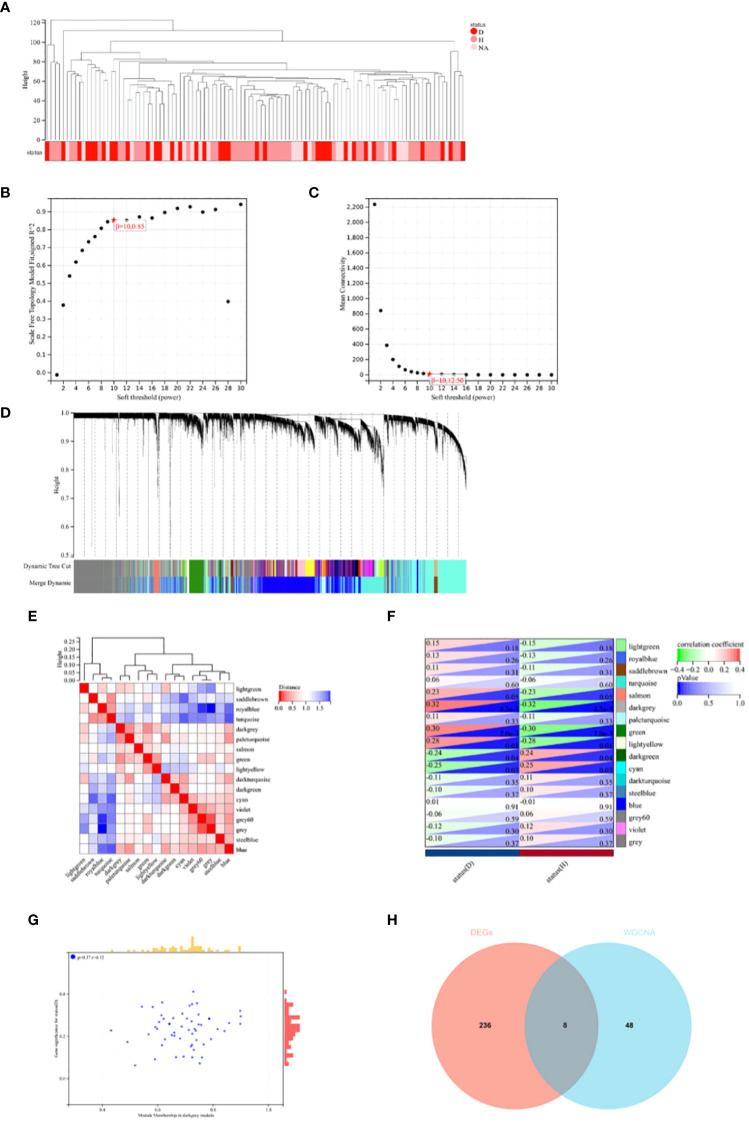
WGCNA Analysis and Venn Diagram. **(A)** In constructing the co-expression network for Intervertebral Disc Degeneration (IDD) and normal conditions, outlier samples were detected through sample clustering analysis; all samples were within the cluster, meeting the cutoff threshold. **(B, C)** Scale-free fit indices for the network topology were obtained through soft-thresholding power analysis. **(D)** Hierarchical clustering analysis was used to detect co-expression clusters with specific color assignments, each color representing a gene co-expression network module constructed by WGCNA. **(E)** A heatmap displays the Topological Overlap Matrix (TOM) for genes used in the weighted co-expression network analysis, with colors ranging from light to red indicating low to high overlap. **(F)** The relationship between two traits and 17 modules in the analysis of significant modules for IDD is shown. **(G)** In the darkgrey module, a scatter plot illustrates the relationship between module membership (MM) and gene significance (GS). **(H)** A Venn diagram shows the intersection of differentially expressed genes (DEGs) with genes from the darkgrey module in WGCNA.

In this study, Weighted Gene Co-expression Network Analysis (WGCNA) was utilized to identify gene modules potentially related to Intervertebral Disc Degeneration (IDD). Initially, all candidate genes and samples were screened to confirm the absence of abnormally high proportions of missing values. Subsequently, through sample clustering analysis, evident outlier samples were excluded with a height cutoff value set at 140 (see **Figure 3A**). The “sft$powerEstimate” was used to determine the optimal soft threshold, selecting 10 as the soft threshold (scale independence reached 0.85) to better differentiate between IDD and normal tissues (see **Figures 3B, C**). With a soft threshold of 10, a minimum module size of 30, and a deepSplit parameter of 3, 17 gene co-expression modules were ultimately identified (see **Figure 3D**). By drawing the inter-module relationship map (see **Figure 3E**) and analyzing the module-IDD feature association using Spearman correlation coefficients, the darkgrey module was found to have the most significant correlation with IDD (see **Figure 3F**). Additionally, a significant correlation was observed between the module membership (MM) and gene significance (GS) within the darkgrey module (see **Figure 3G**). Within the darkgrey module, 56 genes important for module function were identified. Through Venn diagram analysis, co-expressed genes between the differentially expressed genes (DEGs) and the WGCNA-derived feature module genes were identified (see **Figure 3H**).

### 3.4 Identification of characteristic genes and application of machine learning algorithms

**Figure 4:**
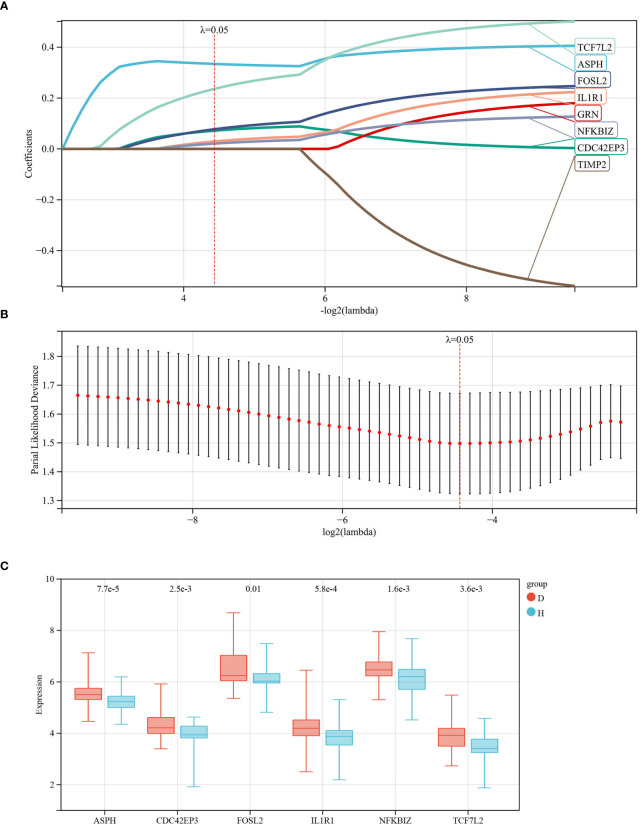
Identification of Characteristic Genes for IDD. **(A, B)** Characteristic genes were obtained through LASSO logistic regression. We set the Lambda value to 0.0462682649698763, ultimately identifying 6 genes; **(C)** Box plots represent the expression levels of the 6 characteristic genes in the database.

Using the R package glmnet, combined with survival time, status, and gene expression data, characteristic genes were identified through lasso-cox regression analysis. A 5-fold cross-validation was utilized to determine the optimal model, selecting a Lambda value of 0.0462682649698763, ultimately identifying 6 genes: ASPH, CDC42EP3, FOSL2, IL1R1, NFKBIZ, TCF7L2 (see **Figures 4A, B**). To visually present the expression levels of these characteristic genes, box plots were drawn, showing an upward trend for these 6 genes in IDD samples compared to control groups (see **Figure 4C**).

### 3.5 Construction of PPI networks to identify core genes

**Figure 5:**
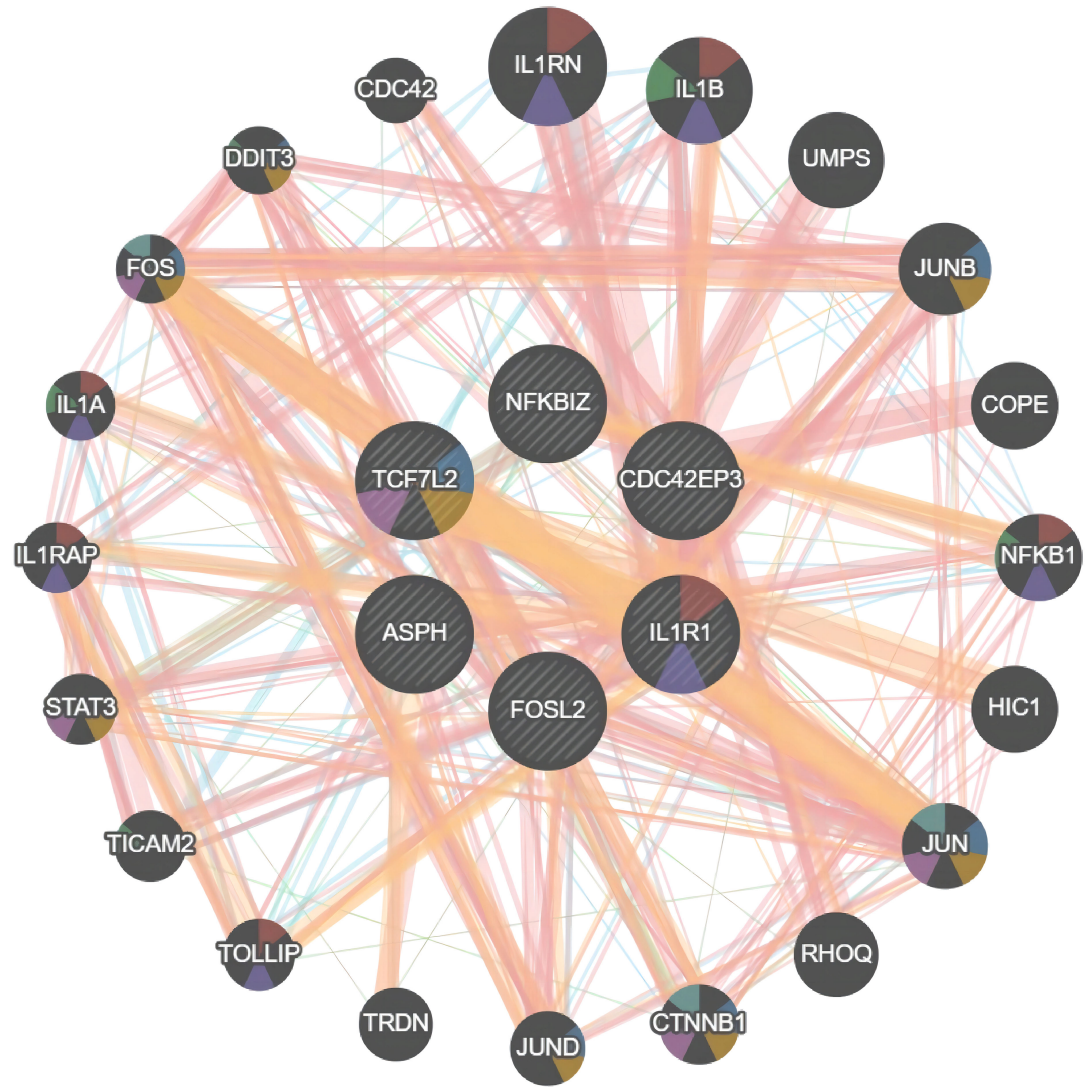
PPI Network Relationship Analysis. Protein-Protein Interaction (PPI) network of ASPH, CDC42EP3, FOSL2, IL1R1, NFKBIZ, TCF7L2 proteins.

Using the GeneMANIA database, we analyzed the Protein-Protein Interaction (PPI) network composed of six genes, ASPH, CDC42EP3, FOSL2, IL1R1, NFKBIZ, TCF7L2, and their interacting genes (see **Figure 5**). In this network, core genes are represented by circles at the center. Around these core genes are 20 circles, representing genes closely related to them, determined based on physical interactions, predictions, biological pathways, and genetic interactions. Through a comprehensive analysis of the PPI network, IL1R1 and TCF7L2 were identified as the final core genes of this study.

### 3.6 Evaluation of the clinical diagnostic value of core genes

**Figure 6:**
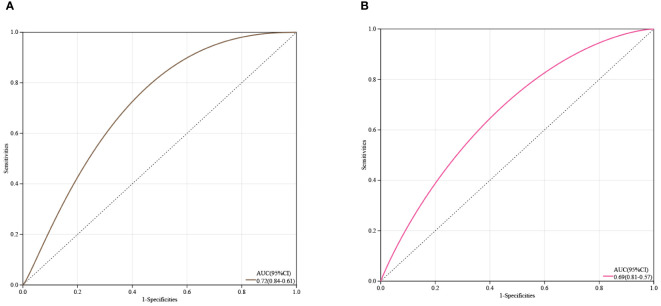
Estimation of the Diagnostic Value of Core Genes. **(A)** ROC curve analysis for IL1R1; **(B)** ROC curve analysis for TCF7L2.

Receiver Operating Characteristic (ROC) curve analysis was conducted on two core genes, IL1R1 and TCF7L2, to estimate their clinical diagnostic value. As shown in **Figures 6A, B**, the Area Under the Curve (AUC) values for both genes were close to 0.7, It is suggested that IL1R1 and TCF7L2 have certain clinical diagnostic value.

### 3.7 Expression validation of core genes

**Figure 7:**
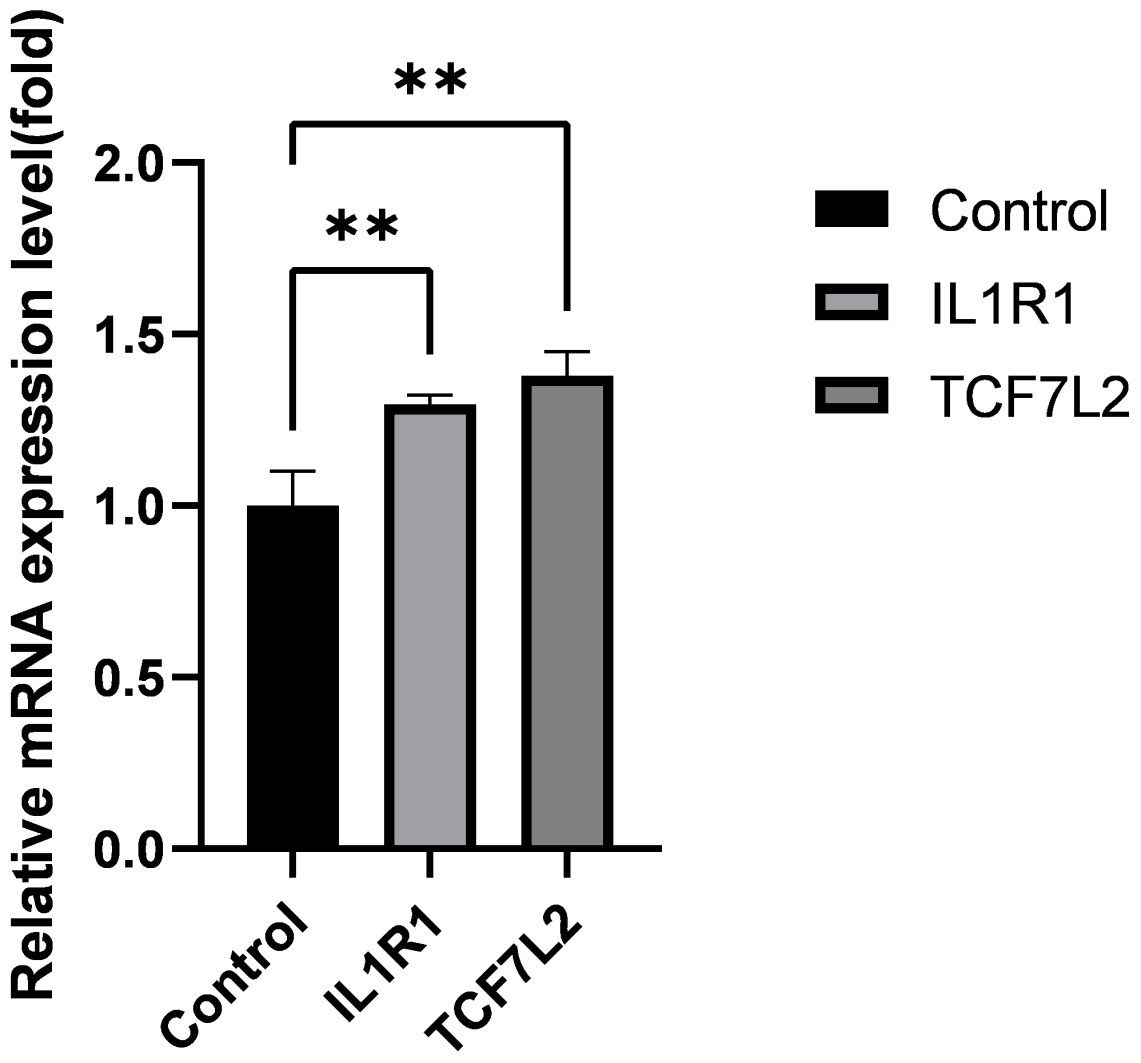
Expression levels of IL1R1, TCF7L2 genes. Data are presented as mean ± SD, **P < 0.01, compared to the control group; assessed by t-test.

Using real-time quantitative polymerase chain reaction (qRT-PCR) analysis, we validated the expression levels of two core genes, IL1R1 and TCF7L2, in the Intervertebral Disc Degeneration (IDD) group compared to the normal intervertebral disc group. The results indicated that the expression levels of IL1R1 and TCF7L2 were significantly higher in the IDD group than in the normal group (see **Figure 7**).

## 4 Discussion

Intervertebral Disc Degeneration (IDD) is a significant cause of lower back pain, imposing a substantial burden on society and the economy. Globally, approximately 700 million patients are affected, with lower back pain not only impacting the physical and mental health of patients but also causing economic losses by reducing social productivity (14). Therefore, studying the pathological changes and molecular mechanisms of IDD is crucial for improving clinical diagnoses and treatment methods. This study delves into the molecular mechanisms of IDD through bioinformatics analysis and machine learning algorithms.

Through differential analysis, we identified 183 upregulated and 61 downregulated genes from four expression profiles obtained from the GEO database, and these differentially expressed genes (DEGs) underwent GO and KEGG enrichment analyses. These genes were enriched in biological processes (BP), cellular components (CC), and molecular functions (MF), unveiling key biological pathways involved in the progression of IDD. WGCNA analysis further produced 17 co-expressed modules, among which the dark grey module showed the highest relevance to IDD, a finding confirmed by the correlation analysis between module membership (MM) and gene significance (GS). Through LASSO regression analysis and PPI network analysis, IL1R1 and TCF7L2 were determined as the core genes of this study. ROC curve analysis indicated that these two genes have good clinical diagnostic value.

Moreover, *in vitro* experiments through RT-qPCR validated the expression differences of IL1R1 and TCF7L2 in IDD patients, consistent with the bioinformatics analysis results.

IL1R1, also known as interleukin-1 receptor type 1, is a gene belonging to the cytokine receptor family of the interleukin-1 receptor family. The protein encoded is a receptor for interleukin 1α, interleukin 1β, and the interleukin 1 receptor antagonist, playing a pivotal role as a mediator in many cytokine-induced immune and inflammatory responses.The pain caused by intervertebral disc degeneration (IDD) is associated with the interaction among nucleus pulposus cells, annulus fibrosus cells, and immune cells such as macrophages, T cells, and neutrophils, leading to the release of pro-inflammatory cytokines (15). The primary cytokines include TNF-α, IL-1, IL-6, IL-17, IL-2, IL-4, and IFN-γ (16, 17), among which TNF-α and IL-1 are the most extensively studied.Research has shown an increase in the gene and protein production of the interleukin-1 receptor type I (IL1R1), rather than the TNF receptor type I, in degenerative intervertebral discs (IVD). Thus, although both cytokines may be involved in the pathogenesis of IVD degeneration, IL-1 is likely to play a more significant role than TNF-α (18). The IL-1 cytokines, IL-1α and IL-1β, act as soluble factors that interact with IL-1R1 and, together with IL-1R3, form a trimeric signaling IL-1 receptor complex. The biological effects of IL-1 are primarily inflammatory, activating target cells at the onset of defensive inflammatory responses. These effects include the induction of inflammatory cytokines, the production of toxic reactive oxygen and nitrogen species, prostaglandins, proteolytic enzymes, etc. The expression of IL-1R1 can be positively and negatively regulated by inflammation-related factors and other stress signals (19). Studies have shown that IL-1 is produced in degenerating intervertebral discs. In degenerative discs, an imbalance between IL-1 and its inhibitor IL-1Ra disrupts the maintenance of matrix homeostasis, leading to an increase in degradative enzymes and a decrease in matrix protein gene expression, characteristic of disc degeneration. IL-1 regulates inflammatory responses through IL-1R1, thus playing a significant role in the development of IDD. Therefore, further research into IL-1R1 as a potential biomarker for IDD is warranted (20).

TCF7L2 is a member of the T-cell factor/lymphoid enhancer-binding factor family (TCF/LEF), a protein-coding gene known as one of the most potent risk loci for type 2 diabetes, involved in several fundamental processes including adipogenesis, autophagy, and alternative splicing (21). Chen S et al. discovered that TCF7L2 is overexpressed in degenerative intervertebral disc tissue samples (22). Silencing TCF7L2 improved lipopolysaccharide (LPS)-induced nucleus pulposus (NP) cell proliferation, extracellular matrix (ECM) synthesis, and alleviated LPS-induced NP cell senescence, marking the first demonstration of the miR-1260b/TCF7L2 axis’s role in maintaining the chondrocyte-like phenotype of NP cells under LPS stimulation and in the synthesis of the extracellular matrix by NP cells.Sun J et al.’s findings indicate that the inhibition of TCF7L2 regulated by miR-155, through the p65/NF-κB signaling pathway, suppresses matrix degradation (23). Inhibiting TCF7L2 may hold therapeutic potential for intervertebral disc degeneration (IDD), as related research has shown a close association between TCF7L2 and the development of IDD.

Nonetheless, this study has limitations, such as the *in vitro* experiments only validating the mRNA levels of the core genes without confirming protein levels through western blot. Therefore, more *in vivo* and *in vitro* experiments are needed to elucidate the roles and potential mechanisms of these two core genes in IDD.

In summary, this study applied bioinformatics and machine learning methods to analyze IDD’s differential genes, including functional enrichment, WGCNA, LASSO regression, PPI network, and ROC curve analyses, and validated the mRNA expression of IL1R1 and TCF7L2 through RT-qPCR experiments, consistent with bioinformatics results. This provides new insights into the pathogenesis of IDD and aids in identifying therapeutic targets.

## 5 Conclusion

This study comprehensively analyzed the pathogenesis of Intervertebral Disc Degeneration (IDD) and identified potential target genes, IL1R1 and TCF7L2, closely related to the progression of IDD. These findings not only enhance our understanding of the mechanisms underlying IDD but also provide potential therapeutic targets for the early diagnosis and treatment of IDD, warranting further in-depth research.

## Data availability statement

The datasets presented in this study can be found in online repositories. The names of the repository/repositories and accession number(s) can be found in the article/supplementary material.

## Ethics statement

The studies involving humans were approved by Ethics Committee of Affiliated Zhongshan Hospital of Dalian University. The studies were conducted in accordance with the local legislation and institutional requirements. The participants provided their written informed consent to participate in this study.

## Author contributions

XY: Writing – original draft, Writing – review & editing. HZ: Writing – original draft, Writing – review & editing. SS: Writing – original draft, Writing – review & editing. XH: Writing – original draft. CG: Writing – original draft. ZJZ: Writing – original draft. ZTZ: Writing – review & editing. JG: Writing – review & editing. MZ: Writing – review & editing.
